# Cell geteromorphism in the conditions of persistence of sapronoses causative agents in various environments

**DOI:** 10.3934/microbiol.2019.2.147

**Published:** 2019-05-23

**Authors:** Larisa M. Somova, Boris G. Andryukov, Irina N. Lyapun

**Affiliations:** 1 Somov Research Institute of Epidemiology and Microbiology, 690087, Selskaya St., 1, Vladivostok, Russia; 2 Far Eastern Federal University, Department of Molecular Microbiology690950, Sukhanova St., 8, Vladivostok, Russia

**Keywords:** sapronoses, morphology, ultrastructure and adaptive changes of pathogens, viable, but noncultivated (VBNC-forms) cells, persister-cells, L-transformation, *Yersinia pseudotuberculosis*

## Abstract

The paper discusses the issues of morphofunctional variability of causative agents of sapronoses under stressful environmental conditions. In the current century, sapronoses infections attract more and more attention. Under unfavorable habitat conditions, their pathogens use a strategy for the formation of resting (stable) states: viable but non-cultured cell forms and the persistence of bacteria, which are characterized by reduced metabolism, changes in the morphology and physiology of microorganisms, and termination of their replication. With the formation of resistant forms of bacteria, the possibility of survival of sapronoses causative agents in the interepidemic period, the formation of their antibiotic resistance, which plays an important role in the chronicity of infections, is associated. The literature widely discusses the mechanisms and conditions for the formation of resistant states of pathogenic bacteria, their pathogenetic significance in infectious pathology, whereas the ultrastructural organization and morphological variability of resistant cellular forms, as well as their differentiation, causing the heterogeneity of the pathogens population, are not yet well covered. The emergence of molecular cell biology methods and the discovery of genetic modules of toxin-antitoxin systems revealed a single mechanism for regulating the formation of resistant cellular forms of bacteria. This served as the basis for the development of fundamentally new technologies for the study of the mechanisms for the conservation of the pathogenic potential of resistant cellular forms of pathogens of natural focal sapronosis in interepidemic periods. Based on the analysis of current data, as well as their own experience, the authors assess the role of morphofunctional changes in resistant cellular forms of bacteria and their significance in the adaptation strategies of causative agents of sapronoses (on the example of *Yersinia pseudotuberculosis*). The study of the manifestations of heteromorphism of causative agents of sapronoses forms the paradigm of the need to improve methods for detecting resistant forms of these bacteria in human and animal biomaterial in order to diagnose chronic recurrent and persistent infections, create effective strategies for monitoring and monitoring the environment.

## 1. Introduction

By the end of the twentieth century, new information was accumulated on the adaptation mechanisms of infectious disease pathogens and the strategies for their existence in unfavourable environmental conditions. To a large extent, this applies to causative agents of sapronoses that can inhabit both in humans and warm-blooded animals, and in environmental objects.

In accordance with the paradigm of interepidemic existence of causative agents of sapronoses infections, two phases of their existence are distinguished: saprophytic and parasitic [1]–[4]. Despite the long period of studying this group of bacteria, more and more information appears on their various adaptation strategies that underlying environmental plasticity of these microorganisms.

At the end of the twentieth century, the stable (‘dormant’) cell forms in non-sporeforming pathogens of sapronoses inhabiting in soils and reservoirs, viable but non-cultured cells (VBNC), and also bacterial persister cells (from English–‘persister’–stable) in the body of warm-blooded animals and people were characterized [5]–[9].

The specific feature of resistant cellular forms of bacteria lies in their low metabolic and replicative activity, which complicates their detection by traditional microbiological methods [10]–[13]. These forms are of great importance in the implementation of the biological properties of a large and diverse group of causative agents of sapronoses: environmental plasticity, variety of resistance forms to external stressors, formation of resistance to antibiotics and other antibacterial agents [1],[13]–[15].

In recent years, a surge of scientific interest in resistant forms of pathogens is associated with the increasing medical and epidemiological significance of the phenomenon of resistant cellular forms and morphological proximity with the already studied and well characterized phenomenon of the bacterial L-transformation [16],[17].

In addition, the development of molecular-cell biology methods and the recent discovery of genetic modules of toxin-antitoxin systems (TAS) revealed a single mechanism for regulating the formation of resistant cellular forms of bacteria [18]–[20]. This served as the basis for the development of fundamentally new technologies for the study of the mechanisms for the conservation of the pathogenic potential of resistant cellular forms in the causative agents of natural focal sapronoses in interepidemic periods [15],[21]–[23].

The border position of this peculiar and extensive group of bacteria capable of both parasitic and saprophytic existence has led to insufficient knowledge of the ways and methods of reserving pathogens, as well as the importance of the morphological signs of resistant cellular forms causing the heterogeneity of populations [2],[24]–[26]. It is possible that the disclosure of specific morphofunctional signs of resistant forms of bacteria will become the missing link in the study of general strategies for the survival of pathogens of natural focal sapronoses both in humans and animals, and in the external environment.

The purpose of this report is to analyze the available data and new information on the role of morphofunctional changes in resistant cellular forms in bacteria and their significance in the adaptation strategies of causative agents of sapronoses.

## 2. Ultrastructural organization of bacteria

There is no doubt that the ultrastructural organization of pathogens characterizes the physiological state of bacterial cells in different habitats. For a long time, ideas about the morphology of bacteria were based on data obtained when they were cultivated in a thermostat at a temperature of 37 °C [27],[28], which characterized the state of pathogens only in warm-blooded human or animal body. The study of the ultrastructure of sapronoses causative agents (*Yersinia pseudotuberculosis, Listeria monocytogenes*) in various trophic and temperature conditions of cultivation [28] allowed us to identify their morphofunctional state not only in the parasitic phase of existence, but also in the saprophytic phase—in the environment. The morphological changes of bacteria were evaluated during their periodic cultivation and during long-term habitat in the soil.

### 2.1. Ultrastructure of sapronoses causative agents in model microecosystems

As is known, the periodic culture of microorganisms is actually a model close to the natural conditions, since in any of its phases the bacteria are in a state of restructuring their metabolism in accordance with changing parameters of the habitat [1],[29]. In experiments with periodical cultivation of bacteria [28], a medium rich in nutrients medium limited in the main bioelements of nutrition were used at temperature conditions creating conditions close to a warm-blooded organism or the environment.

Studies in model microecosystems (periodic and soil cultures of *Y. pseudotuberculosis* and *L. monocytogenes*) provided an idea of the adaptive variability in populations of causative agents of saprozoonoses in their characteristic, changing habitat conditions [16],[28]. Both under the conditions of the parasitic phase of existence (periodic cultivation) and under the conditions of the saprophitic phase (soil cultures), the similar morphological changes of adaptive nature were established, namely: the formation of cytoplasmic outgrowths (prostakes), the accumulation of reserve substances, increased tortuosity and change in cell wall thickness, changes in the ribosomal saturation of the cytoplasm and the state of the nucleoid associated with a conformational change in bacterial DNA. All these changes are aimed at preserving populations of causative agents of sapronoses in various habitats [28].

### 2.2. Pathogenic bacteria under stress

An important stage in the development of ecological and epidemiological trends in microbiology has become research relating to the study of bacterial populations under stressful environmental conditions, in which bacteria are able to enter a state of rest with a severe decrease in their metabolic activity and temporary loss of their ability to reproduce [1],[30]–[32]. It has been established that bacteria have original ways to survive during stress [33]–[35], for example, caused by the inevitable depletion of nutrients, as well as the effects of antibiotics. Two different phenotypes are described in which cells enter into a non-inherited, reversible, inactive state: viable, but non-culturable (VBNC) cells and persistent cells [7],[14],[15],[36]. The Persistent cells (persister-cells) were discovered as early as 1942 Hobby et al. [37], who established that 1% of cells in the population of *Staphylococcus aureus* were not killed by high doses of penicillin and were characterized by metabolic and replicative dormancy.

Forty years later, VBNC cells were first described in *Escherichia coli* and *Vibrio cholerae*, appearing after a long period (two weeks) in saltwater microcosms, but non-culturable in the selective and accepted media in which they are usually capable of growing, but several stimuli such as nutrients and temperature shifts, lead to the recultivation of VBNC forms [8],[13],[38],[39].

The two resting states of bacteria revealed a lot in common. Both persistеr-cells and VBNC forms are associated with chronic infections, both conditions of bacteria are present in biofilms [15],[40]–[42], and also form under more than one type of stress, for example, oxidative or acidic [15],[43]. The genetic basis for both cell types has not been well characterized. The role of toxin-antitoxin systems (TAS) in the induction of the VBNC state is described [7],[18]–[20]. It is reported that these systems, which are classically involved in the formation of the cell wall, also induce the formation of persistent cells during incubation in human serum [7],[15],[44], which has clinical significance [45].

The most important role in the formation of persistent cells is played by guanosine tetraphosphate (ppGpp) [39] and the sigma factor of the stationary phase RpoS [9]. RpoS [8],[43], the OxyR transcriptional regulator that controls genes associated with oxidative stress [15], and TAS [7],[18]–[20] are associated with VBNC cells. Consequently, it has been suggested that these two states of bacterial survival may be part of a ‘rest continuum’ [7],[15],[20].

The key feature that distinguishes persister-cells from VBNC forms is that they cannot be reanimated (recultivated) under normal conditions, while persisters can easily be converted to a vegetative state sensitive to antibiotics or other stresses [21]. VBNC forms and persister-cells have many similarities, and they can coexist [20],[32],[38], but the authors have not conducted any studies to compare these two stages of resting cells in their physiology and morphology.

So, now it is proved that bacteria have two resistant phenotypes: VBNC and state of persistence. Both resting forms occur without mutation, and both are associated with chronic infections; however, a subpopulation of persistent cells is capable of rapid recultivation upon the occurrence of favorable growth conditions, while cells that are in a state of VBNC can be in this form for years. VBNC-forms were present in experimental samples with isolation of persisters, which again confirms the fact that these cellular forms coexist, are induced by the same conditions and are regulated by TAS [12].

A recent study [28] traced the relationship between these two stress-induced phenotypes of bacteria using transmission electron microscopy and fluorescence microscopy with the study of cell morphology and quantitative determination of reanimated (surviving) cells. The authors found that the viable proportion of VBNC cells formed resulting nutrient depletion is represented by persistent cells based on a comparison of their antibiotic tolerance, reanimation rate, morphology, and metabolic activity. The rest of the cell fraction VBNC was not viable. The authors concluded that the phenotype of a resting cell, known as VBNC, is the same, known as persistent cells.

As already mentioned, the state of resistance in enteropathogenic bacteria is expressed in the temporary loss of the replicative and metabolic activity of microorganisms [12],[14],[15],[20]. Phenomenon of resting states in bacterial cells is of great importance in the chronization of the infectious process in humans and animals, since it has been established that resistant forms are able to reanimate in vivo and restore their virulence [13],[31],[36].

### 2.3. Dormant forms of bacteria as infectious agents

Bacteria constantly encounter with the problems of potentially dangerous environmental uncertainty and, to avoid such constant instability of their existence, many microorganisms maintain subpopulations with the possibility of transition to a temporary state of rest, during which cells show a decrease in growth rates and metabolic activity [33]. When the environment becomes favorable, resting cells can be reanimated and subsequently to restore the growth [7],[15],[21]. The evolutionary role of maintaining such heterogeneity of the bacterial population is due to the fact that the emergence of different cells phenotypes increases the probability of pathogen survival in an unstable environment [33].

It is important to note that the resting state, which allows bacteria to confront environmental stress, can also make them tolerant to antibiotics [23],[36],[44], which emphasizes the clinical significance of this physiological state. It has been found that at least 85 species of bacteria enter the VBNC state [15]. This condition was alternatively referred to by other authors as conditionally viable ecological cells (CVEC) [9], active but non-cultured cells (ABNC) [22] and dormant cells [45]. It turned out that these cells are viable due to their intact cell membrane, low-level metabolic activity and the continuation of gene expression [31],[36]. The state of VBNC is considered an effective survival strategy for the bacterium, as it allows cells to withstand unfavourable environmental conditions and reanimate the replicative form while improving environmental conditions.

Thus, bacterial cells in a state of VBNC appear to be the same as persistent cells based on antibiotic resistance, morphology, reanimation rate, and metabolic activity. To date, it has been suggested [14] that the terms ‘VBNC’ and ‘persisters’ describe the same phenotype for resting (dormant) cells and that the term VBNC should be replaced by persistent cells, since VBNC cells are not a separate phenotype.

Attracts attention that most of the studies on this problem are devoted to the study of the detection of non-cultivated forms and the induction of VBNC state in causative agents of bacterial infections, while the ultrastructural organization and morphological variability of VBNC and persistent cells are still not well covered.

In Russia, the intensive research on the study of uncultivated forms of causative agents of sapronoses was carried out in the N.F. Gamaleia Institute of Epidemiology and Microbiology RAMS in the 1980–1990s. Based on the results of the studies, the presence of VBNC forms in *Yersinia* and *Listeria* was found in experimental animals and soil populations of bacteria [1]–[3],[26]. The authors concluded that these forms are important for the reservation and adaptation of pathogens to unfavorable environmental conditions.

When studying the ultrastructure of sapronoses causative agents in various environmental conditions [28], attention was paid to the morphology of *Y. pseudotuberculosis* with long-term habitat in the soil and with periodic cultivation in the stationary phase / the die-off phase, when bacteria can become non-cultivated.

It should be noted that during periodic cultivation of *Y. pseudotuberculosis*, the number of bacterial populations and the period of onset of the die-off phase depended on trophic and temperature factors. So, according to Somova et al. [28]. The largest population of *Yersinia* in the stationary phase was observed when cultivated at a temperature of 18–20 °C, the death of bacteria began after 3 days. When the cultivation temperature was 37 °C, the death of the bacteria began after 15 days. Since Y. pseudotuberculosis has psychrophilic properties, the cultivation temperature of 6–8 °C is most favorable for them, during which the active proliferation of bacteria (exponential phase) lasted up to 5 days, and the death phase of bacteria was not observed during the entire observation period (40 days) [28].

Taking into account the dynamics of reproduction of *Y. pseudotuberculosis* in different nutrient conditions, attention was paid to the appearance of dormant bacterial forms grown at different temperatures (37, 18–20 and 6–8 °C) in the dying off phase. Changes in the spheroplastic type were revealed in these cell forms: signs of partial lysis with dark areas of the cytosol, but the cell wall and the main ultrastructural intracellular elements remained. The nucleoid in such cells had the appearance of coarse chromatin fibrils and agglomerates, while in the nucleoid zone, bacterial cells with electron-dense chromatin fibrils remained.

**Figure 1.**
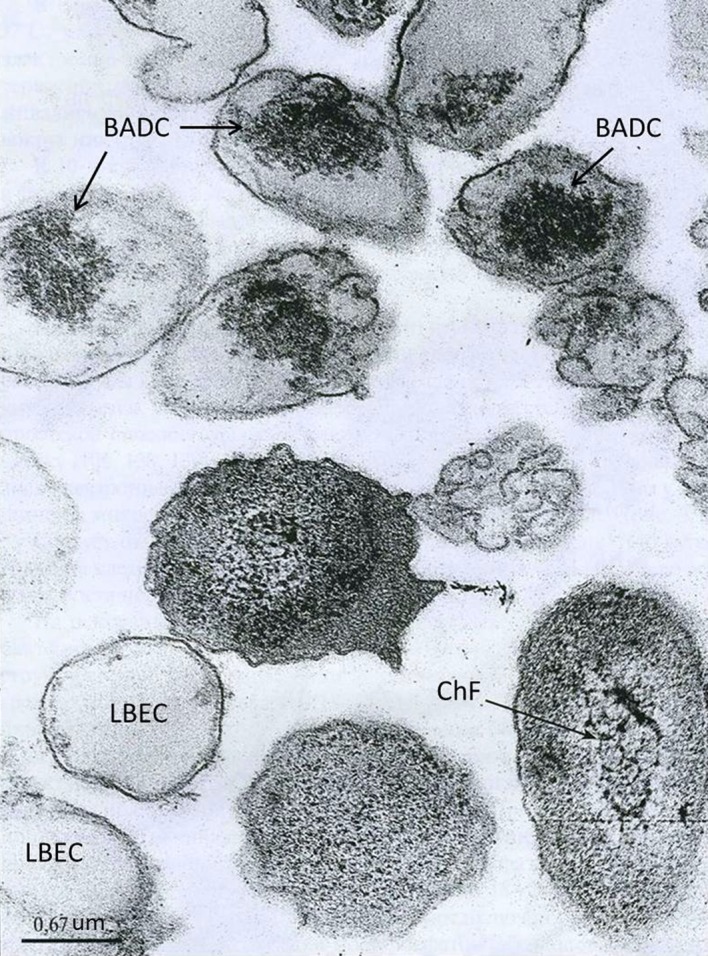
The *Y. pseudotuberculosis* periodic culture in the stationary phase/the die-off phase [28]. Designations: LB: lysed bacterium; BADC: bacteria with areas of dense cytosol; ChF: chromatin fibrils in the nucleoid zone.

**Figure 2.**
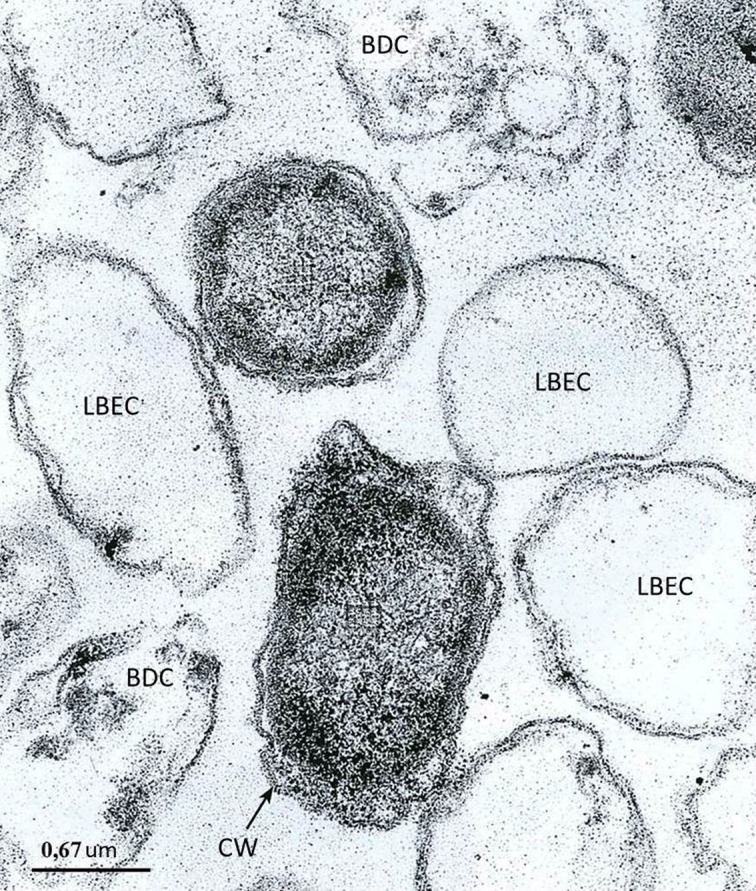
The *Y. pseudotuberculosis* periodic culture in the stationary phase/the die-off phase [28]. Designations: CW: cell wall; LBEC: lysed bacterium with empty cytosol; BDC: bacteria with dense cytosol and cell wall detachment.

Thus, during the periodic cultivation of *Y. pseudotuberculosis* in the die-off phase, in a bacterial population cells were identified that resembled non-cultuvated forms, similarly to that described by Kim et al. [14]. An empty cytosol in part of the cells is regarded as a sign of their death [14],[30]; therefore, we can assume that the population of *Y. pseudotuberculosis* in the die-off phase contains VBNC cells, some of which were not viable (Figures 1 and 2).

### 2.4. Heteromorphism of Yersinia pseudotuberculosis in soil habitat

The study of the morphofunctional state of *Y. pseudotuberculosis* when habituating in soil at different temperatures was carried out in long-term experiments on model microecosystems—a soil reservoir and flow-through soil columns [28].

Three months later, the soil population of *Y. pseudotuberculosis* consisted mainly of ovoid-shaped bacteria, but occasionally isolated giant cells were divided into several irregular segments. A rarefied nucleoid zone was detected in bacterial cells. Some of these bacteria were in a state of binary division. A fine granular component remained on the outer membrane of the cell wall.

Bacterial cells of a 7-month soil culture were distinguished by significant heteromorphism. A large number of deformed bacteria attracted attention; however, they, as a rule, did not have changes in the spheroplastic type, retaining the main ultrastructures. Thinning of the cell wall was observed in such cells, in some of them the nucleoid was not detected. The surface of bacterial cells often had a scalloped appearance due to the formation of prostakes, which increase the surface of the cell wall and, accordingly, the consumption of nutrients. The appearance of fibrillar electron-dense chromatin structures, having a curl shapes and parallel to each other, attracted the attention. Some bacterial cells had up to 2–3 such structures, reflecting the conformational changes in DNA [14],[27],[28]. Similar chromatin condensation zones were also found in dividing bacteria in the region of the formation of transverse waist. Heteromorphism of cells in the cytosol ribosomal saturation was also observed, which indicated differences in metabolic activity in cells of the bacterial population. The ribosomes and polyribosomes were practically not detected; to a greater or lesser extent the cell was filled with ribonucleoprotein agglomerates [28].

In samples of a 9-month soil culture of *Y. pseudotuberculosis*, the bacterial cells had a significant rarefaction and defects in the nucleoid zone. The colonization of cells was observed by means of an intercellular amorphous matrix tightly connecting them with each other. Intercellular bridges could be seen between the bacteria. Bacteria-revertants of this culture were similar in size and ultrastructure to bacteria of a one-month soil culture, but in the area of the nucleoid they retained coarse chromatin fibrils.

Thus, when habitating under the conditions of a soil reservoir, exposed to weather conditions, the *Y. pseudotuberculosis* bacteria underwent changes in the morphofunctional state. Apparently, these changes were adaptive in nature and indicated a high ability to adapt the causative agent of *Y. pseudotuberculosis* infection to unfavorable environmental conditions.

When habitating in soil, a common feature for *Y. pseudotuberculosis* strains was a temperature-independent increase in the number of bacteria with a thickened cell wall, with an increase in the observation period. The cell wall thickness in the two studied bacterial strains increased on average to 830 Å compared to the control (75 Å). By the two years of observation, this feature was characteristic of all bacteria of the studied soil variants of *Y. pseudotuberculosis*. The bacteria of the *Y. pseudotuberculosis* culture (strain H-2781), which had been in soil columns for three months at a temperature of 6–8 °C, were represented mainly by rounded (coccoid) and, in a smaller amount, ovoid forms. At all periods of observation, the presence of coarse fibrils and chromatin agglomerates was characteristic of bacteria of ‘cold’ cultures. As noted above, the chromatin condensation in a bacterial cell indicates that DNA is in the composition of the nucleoprotein and is best protected from external influences [1],[2]. Therefore, it can be assumed that the electron-dense structures of nucleoproteins are the morphological sign of causative agents of sapronoses in the saprophytic phase of existence.

Based on the above, it can be concluded that, when habitating in open-type soil ecosystems, ultrastructural changes occur in the studied bacteria (capsule and microcapsule formation, total cover, mucus, intercellular contacts, prostakes, reserve substances, increased tortuosity and change in cell wall thickness, changes in cell size , ribosomal saturation of the cytoplasm and the state of DNA), contributing to the survival of bacterial cells under constant exposure to biotic and abiotic factors of the environment. Such changes are characteristic of R-and transitional forms of bacteria [11],[14],[20],[26], [28].

Thus, it can be assumed that the appearance of a changes complex in the ultrastructure of *Y. pseudotuberculosis* bacteria should be regarded as a natural adaptive response to changing habitat conditions in the corresponding ecological niche. The appearance in periodic and soil cultures of bacteria the similar changes in ultrastructures that perform the same function in the bacteria indicates the universality of adaptation mechanisms.

### 2.5. L-transformation as a form of bacteria persistence

L-transformation of bacteria is considered as one of the important factors creating the possibility of pathogens persistence and recurrence of infectious diseases [1],[2],[16],[17]. In these studies, the L-transformation of bacteria was characterized as a regular peculiar form of their adaptation to the changed conditions of the habitat. At the moment, the unambiguity of this phenomenon and other manifestations of the bacteria heteromorphism is not determined. It is known that L-transformation is characteristic of many bacteria and in vivo can occur under the influence of various endogenous factors, primarily lysozyme, lysosomal enzymes and amino acids. Bacterial enzymes of phagocytes that affect the phospholipids and peptidoglycans of bacterial walls have a damaging effect on bacteria [17].

In 7–14 days after infection in animals, infected with virulent *Y. pseudotuberculosis* strains, in the organs, along with typical and destructively altered bacterial cells, forms of protoplastic and spheroplastic types were detected [16] with the presence of myelin-like structures around bacteria, similar to those in cell culture, infected with *Y. pseudotuberculosis* [27]. The formation of such structures is most often associated with lipid peroxidation of cell membranes under the influence of various damaging factors. Similar changes in the ultrastructure of *Y. pseudotuberculosis* were also noted when its interacting with infusoria [26], and it was suggested that bacteria surrounded by myelin-like membranes may remain viable for a long time. In our opinion, the formation of myelin-like structures characterizes the picture of incomplete phagocytosis peculiar to *Y. pseudotuberculosis* infection and some other infections caused by facultative intracellular bacteria, which also agrees with the data of Pushkareva et al. [26] in regard to bacteria of the *Yersinia* genus. It is possible that the identified in experimental studies the ultrastructural changes in *Y. pseudotuberculosis* are in close connection with changes in the virulence of bacteria during infection.

Further studies of the morphofunctional variability of pathogenic bacteria will help to come closer to solving the sacramental issue of academician Somov: where, how and when the virulence of causative agents of sapronoses recovery occurs in the environment, initiating the emergence of the epidemic (epizootic) and infectious processes [4].

The uncovering new mechanisms for the induction of an non-cultured state in causative agents of sapronoses in the parasitic and saprophytic phases of their existence, as well as identifying previously unknown conditions for the reversal of non-cultivated forms into vegetative forms determining the development of the infectious process, has the fundamental and applied importance. An indepth study of the heteromorphism manifestations of bacteria under stressful conditions should be aimed at improving the methods for detecting non-cultivated forms of the causative agents of sapronoses in biomaterial from humans and animals in order to diagnose chronic-recurrent and persistent infections.
